# Advances in Functional Genomics and Biotechnology for Enhancing Therapeutic Potential of Medicinal Plants

**DOI:** 10.3390/ijms27104245

**Published:** 2026-05-10

**Authors:** Wajid Zaman, SeonJoo Park

**Affiliations:** Department of Life Sciences, Yeungnam University, Gyeongsan 38541, Republic of Korea

**Keywords:** RNA sequencing, functional genomics, CRISPR/Cas9, metabolic engineering, cannabinoids, personalized medicine

## Abstract

Medicinal plants have long served as a primary source of bioactive compounds with essential therapeutic applications. Recent advances in functional genomics and plant biotechnology now enable precise manipulation of metabolic pathways to enhance the production of specialized metabolites with medicinal value. However, an integrative understanding of how genomic discovery can be linked with pathway engineering, scalable production systems, and healthcare applications remains insufficiently developed. This knowledge gap limits the effective translation of molecular insights into the sustainable production of medicinally important compounds. The novelty of this review lies in its integrated framework linking functional genomic discovery with pathway engineering, synthetic biology, artificial intelligence-assisted prediction, and scalable production systems for medicinal plant-derived therapeutics. This review aims to provide a comprehensive overview of cutting-edge approaches in medicinal plant research, emphasizing high-throughput RNA sequencing, CRISPR/Cas9 gene editing, synthetic biology, and metabolic engineering for optimizing the production of key bioactive compounds, including artemisinin, cannabinoids, ginsenosides, and taxol. It further examines how these tools collectively support metabolite discovery, pathway elucidation, yield improvement, and biotechnological production in major medicinal plant systems. We explore the application of genomic and biotechnological approaches in plants such as *Artemisia annua*, *Cannabis sativa*, *Panax ginseng*, and *Taxus baccata* to enhance metabolite yields and promote sustainable production. The review highlights case studies that demonstrate how genetic modification, metabolic engineering, and synthetic pathway design have been successfully employed to increase the synthesis of key medicinal compounds. Moreover, we discuss the integration of artificial intelligence and machine learning to predict gene–metabolite relationships, support personalized phytochemical therapies, and facilitate sustainable, large-scale production. Finally, the review addresses the implications of these innovations for the pharmaceutical industry, healthcare, and agriculture, while also highlighting sustainable and scalable directions for future medicinal plant biotechnology.

## 1. Introduction

Medicinal plants have played a central role in human healthcare for centuries. They serve as the foundation of traditional medicine across various cultures and continue to provide a rich source of therapeutically effective compounds [1]. Many important drugs, including morphine, quinine, and aspirin, have been derived from plant sources [2]. The importance of these plants is further highlighted by the fact that ~25% of modern medicines are derived from plant-based natural products, which remain the primary treatment for many diseases in developing regions [3]. The increasing global reliance on these plants for healthcare, alongside the growing interest in natural and sustainable therapeutics, has created an urgent need to understand the complex biological systems underlying these plants. Consequently, medicinal plants remain integral to traditional and modern therapeutic practices [4].

Specialized metabolites, also referred to as secondary metabolites, are bioactive compounds produced by plants that largely underlie their medicinal properties [5]. Unlike primary metabolites involved in fundamental cellular processes, these compounds have evolved in plants to perform functions such as defense, protection from UV radiation, and interactions with the surrounding environment, including other organisms [6]. Secondary metabolites, such as alkaloids, terpenoids, and flavonoids, exhibit diverse pharmacological activities, ranging from anti-inflammatory, anticancer, and antiviral properties to analgesic and antioxidant effects [7]. The structural diversity and biochemical complexity of these metabolites make them the focus of research, as they represent a vast and largely untapped reservoir for new drug development. However, the systematic study and production of these metabolites remain challenging due to their complex biosynthetic pathways and the variation in their accumulation across plant species and environmental conditions [8].

Functional genomics has become a pivotal approach for dissecting the complexity of plant specialized metabolite pathways. Using high-throughput techniques such as next-generation sequencing (NGS) and RNA sequencing (RNA-seq), the complete genomic landscape of medicinal plants can now be characterized, enabling the identification of genes and regulatory networks that govern metabolite biosynthesis [9]. Functional genomics allows the identification of specific genes involved in the biosynthesis of bioactive compounds, providing insights into the genetic basis of medicinal properties [10]. Additionally, gene editing technologies such as CRISPR/Cas9 have enhanced the capacity to modify plant genomes, thereby improving targeted metabolite production [11]. This integration of genomics with biotechnology has accelerated the development of genetically modified plants with enhanced yields of therapeutic compounds [12]. Advances in genomic tools, along with the increasing availability of plant genome sequences, have created new opportunities for efficient metabolic engineering, enabling the targeted manipulation of specific secondary metabolite production [13].

Despite advances in functional genomics, significant challenges remain in understanding the intricate pathways of metabolite biosynthesis, particularly in species that produce rare or hard-to-isolate compounds [14]. The biosynthetic networks of secondary metabolites are frequently shaped by multiple genetic and environmental factors, including epigenetic regulation, hormonal signaling, and microbial interactions [15]. Moreover, the complexity of secondary metabolite pathways complicates the prediction of how genetic modifications will influence the production of target compounds. For instance, many medicinal plants rely on enzyme cascades to produce a final product, and altering a single enzyme can have unintended consequences on other downstream steps, ultimately affecting the yield and quality of the metabolite [16]. In addition, genomic resources for many medicinal plants remain incomplete, numerous biosynthetic pathways are still only partially resolved, and the integration of multi-omics datasets with functional validation remains limited. More importantly, the translation of gene discovery into metabolic engineering, synthetic biology, and scalable production systems has not yet been sufficiently integrated into a clear conceptual framework. These limitations restrict the efficient conversion of molecular knowledge into consistent, high-yield, and sustainable production of medicinally important specialized metabolites [17].

Therefore, this review aims to critically synthesize recent advances in functional genomics and biotechnology for medicinal plants, with a specific focus on how high-throughput sequencing, CRISPR/Cas9-mediated genome editing, synthetic biology, metabolic engineering, and scalable culture systems can be integrated to enhance the discovery, validation, and sustainable production of therapeutically important specialized metabolites. Although numerous studies have identified candidate genes and pathways, the translation of these molecular discoveries into reliable, scalable, and sustainable production systems remains limited. In particular, a clear research gap exists in integrating functional genomics with pathway validation, synthetic biology, metabolic engineering, and artificial intelligence-assisted prediction. By integrating genomic tools, metabolic engineering, and synthetic biology, deeper insights can be gained into the complex pathways governing the biosynthesis of bioactive compounds. This review highlights recent advances in high-throughput sequencing, CRISPR/Cas9 gene editing, and biotechnological applications such as plant tissue cultures and bioreactor systems. Additionally, it presents selected medicinal plants, including *Artemisia annua*, *Cannabis sativa*, *Panax ginseng*, and *Taxus baccata*, as representative systems illustrating how functional genomics can enhance the production of therapeutic compounds. 

## 2. Next Generation Functional Genomics Tools in Medicinal Plant Research

Functional genomics is essential for understanding the molecular mechanisms underlying the production of bioactive compounds in medicinal plants [18]. The advent of NGS technologies has greatly enhanced the scope and resolution of plant genomic research [19]. Using tools such as RNA-seq, the complete transcriptome of a plant species can now be captured, providing critical insights into gene expression, regulatory networks, and metabolic pathways [20]. This approach has created new opportunities to identify genes involved in the biosynthesis of specialized metabolites, which are often challenging to isolate or study using traditional methods [21]. Examining gene expression profiles under varying environmental conditions or stress responses enables the identification of genes responsible for producing compounds such as alkaloids, flavonoids, and terpenoids, thereby facilitating the improvement and engineering of medicinal plant traits [22].

### 2.1. High-Throughput Sequencing and Transcriptomics

RNA-seq is a powerful tool in functional genomics for high-throughput analysis of plant gene expression. This technology enables the simultaneous sequencing of millions of RNA molecules, offering a comprehensive view of the transcriptome [23]. RNA-seq is especially valuable in medicinal plant research, as it can detect low-abundance transcripts that are often crucial for producing medicinal compounds [24]. Unlike microarray-based methods, RNA-seq does not rely on pre-designed probes, making it more flexible and applicable across a wider range of plant species [25]. In medicinal plants, RNA-seq is employed to identify genes involved in the biosynthesis of bioactive metabolites, such as alkaloids in *Papaver somniferum* and flavonoids in *Citrus sinensis* [26]. This approach facilitates the identification of major regulatory genes, thereby improving metabolite optimization of metabolite production for pharmaceutical applications [27].

**Figure 1:**
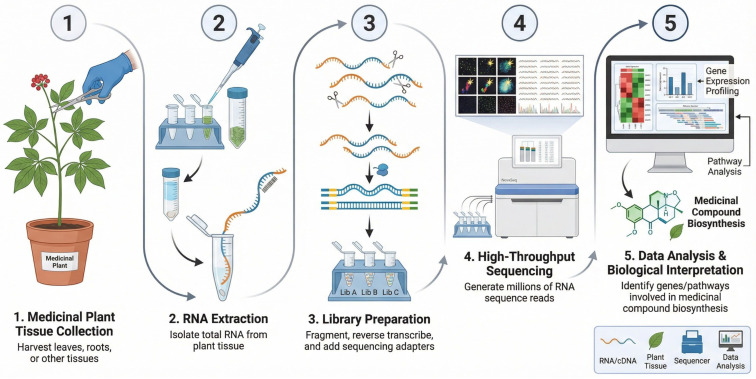
Schematic overview of the RNA-seq workflow in medicinal plant research, including tissue collection, RNA extraction, library preparation, high-throughput sequencing, and downstream data analysis for gene expression profiling and pathway identification related to medicinal compound biosynthesis. Elements in the figure are represented schematically for conceptual illustration only and are not intended to depict detailed molecular structures, reaction steps, stoichiometry, or complete biochemical mechanisms.

A notable application of RNA-seq is the study of terpenoid biosynthesis in *C. sativa* [28]. Terpenoids are a class of secondary metabolites with diverse medicinal properties, including anti-inflammatory, analgesic, and anticancer activities [29]. RNA-seq analysis of different *C. sativa* strains enables the identification of genes involved in cannabinoid biosynthesis, such as tetrahydrocannabinol (THC) and cannabidiol (CBD), and facilitates examination of how environmental factors and genetic variation influence their levels [30]. This approach has yielded detailed insights into the complex terpenoid biosynthetic pathways, which are regulated by networks of genes and transcription factors [31]. Figure 1 illustrates the RNA-seq workflow, from sample collection to sequencing and gene expression analysis, highlighting its application in medicinal plant research. 

### 2.2. Metagenomics and Microbiome Influence on Medicinal Plants

The role of the microbiome in plant health and metabolism is an emerging area of research, especially in medicinal plants. Plants continuously interact with their associated microbiota, which can significantly influence growth, stress tolerance, and metabolic profiles [32]. Key microbiome components, including rhizobacteria and mycorrhizal fungi, have been shown to enhance the production of bioactive compounds in plants [33]. Rhizobacteria can enhance the biosynthesis of secondary metabolites by producing plant growth-promoting hormones or by modulating plant metabolism through nitrogen fixation [34]. For instance, specific rhizobacteria strains in *Coriandrum sativum* (coriander) can stimulate the production of flavonoids and phenolic acids, which are essential for the defense and medicinal properties of the plant [35]. Similarly, mycorrhizal fungi establish symbiotic relationships with plant roots, enhancing water and nutrient uptake while simultaneously modulating the production of terpenoids and alkaloids. This interaction holds significant potential for improving the yield and quality of medicinal plant metabolites [36]. 

Microbial communities can also enhance plant resistance to biotic stresses, such as pathogens, by modulating the immune system and inducing systemic resistance [37]. Metagenomics, the study of genetic material recovered directly from environmental samples, has been especially valuable for characterizing the plant microbiome [38]. Sequencing microbial communities associated with medicinal plants enables the identification of beneficial microbes and provides insights into their roles in plant metabolism. This approach reveals new opportunities for enhancing the phytochemical production through targeted microbiome manipulation [39]. Advances in metagenomic sequencing technologies enable the comprehensive analysis of entire microbial communities, providing deeper insights into the complex interactions between plants and their associated microbiota [40].

### 2.3. CRISPR/Cas9 and Gene Editing Technologies

Developing CRISPR/Cas9 and other gene editing technologies has transformed plant biotechnology, offering unprecedented precision in genome modification [41]. In medicinal plants, these tools are employed to manipulate genes involved in secondary metabolite biosynthesis, leading to increased yields of bioactive compounds. CRISPR/Cas9 enables targeted deletion, insertion, or modification of genes in the plant genome, thereby significantly enhancing or redirecting metabolic pathways [42]. For example, CRISPR/Cas9 has been applied to *A. annua* to improve artemisinin biosynthesis. CRISPR has been used to disrupt genes involved in competing metabolic pathways, thereby redirecting plant resources toward artemisinin production [43]. This strategy has increased artemisinin yields, making production more cost-effective and sustainable than traditional cultivation methods [44].

CRISPR/Cas9 has also been applied to *C. sativa* to enhance cannabinoid production, targeting genes involved in THC and CBD synthesis [45]. Disrupting specific enzymes in the terpenoid biosynthesis pathway enables increased accumulation of desired cannabinoids while minimizing the formation of unwanted by-products [46]. This finding demonstrates the potential of gene editing to optimize the genomic architecture of medicinal plants and maximize their therapeutic potential. The precise genome-editing capability of CRISPR/Cas9 has broad applications in medicinal plant biotechnology, enabling more efficient and sustainable production of therapeutic compounds [12].

### 2.4. Single-Cell Genomics for Specialized Metabolite Mapping

Single-cell RNA-seq is a cutting-edge technique that provides detailed insights into the cell-specific production of specialized metabolites in plants [47]. Unlike traditional genomics approaches, which measure average gene expression across entire tissues, single-cell methods capture the heterogeneity among individual cell types. Single-cell RNA-seq enables analysis of gene expression at the single-cell level, which is particularly valuable for studying metabolite production in specialized plant cell types such as glandular trichomes, phloem, and root cells [48]. For instance, glandular trichomes in C. sativa produce cannabinoids, and single-cell RNA-seq has been used to identify the specific genes responsible for THC and CBD biosynthesis in these cells [49]. This approach offers insights into metabolic processes occurring at the cellular level and supports a more targeted strategy to enhance metabolite production in medicinal plants [50]. Table 1 summarizes the various specialized plant cell types involved in secondary metabolite biosynthesis, highlighting their roles in the biosynthesis of bioactive compounds.

For instance, glandular trichomes in *C. sativa* produce cannabinoids, root cells in *P. ginseng* synthesize ginsenosides, and phloem cells in *Citrus* spp. produce flavonoids [69]. Understanding the cellular specialization of metabolite production supports the optimization of metabolic engineering strategies to enhance the yield of specific bioactive compounds. Integrating single-cell genomics with functional genomics enables a comprehensive understanding of plant metabolism and facilitates the identification of key regulatory genes involved in specialized metabolite biosynthesis [70].

## 3. Specialized Metabolite Pathways in Medicinal Plants

Medicinal plants are abundant sources of specialized secondary metabolites. These compounds are typically produced as defense responses to environmental stressors and simultaneously offer significant benefits to human health [5]. The biosynthesis of these metabolites is governed by complex pathways involving the coordinated activity of multiple enzymes and regulatory proteins. Understanding these pathways is essential for optimizing the production of bioactive compounds in medicinal plants, whether through conventional cultivation or advanced biotechnological approaches [71]. The primary classes of specialized metabolites, including flavonoids, alkaloids, terpenoids, and glycosides, are central to the medicinal properties of plants, with each class exhibiting distinct pharmacological properties [72].

### 3.1. Flavonoids and Phenolic Compounds

Flavonoids and phenolic compounds are among the most extensively studied secondary metabolites because of their potent antioxidant, anti-inflammatory, anticancer, and cardioprotective activities [73]. Flavonoid biosynthesis is tightly controlled by a series of enzymes in the phenylpropanoid pathway, which converts simple precursors into complex flavonoid structures [74]. The pathway begins with the conversion of phenylalanine to cinnamic acid, followed by a series of enzymatic reactions that produce flavonoids, including quercetin, kaempferol, and anthocyanins [75]. Flavonoids are commonly classified based on their chemical structure, with flavonols, flavones, and anthocyanidins representing the most prominent subgroups [76]. In addition to their antioxidant activity, which neutralizes free radicals, flavonoids modulate several biochemical pathways involved in inflammation, cell signaling, and gene expression [77].

An illustrative example of the therapeutic potential of flavonoids involves quercetin in Citrus and catechins in Camellia sinensis [78]. Quercetin, a flavonoid abundant in Citrus fruits, has been extensively studied for its anti-inflammatory and antioxidant activities [79]. Evidence indicates that quercetin may reduce the risk of chronic diseases, such as cardiovascular disease and diabetes, via modulating oxidative stress and improving endothelial function [80]. Similarly, catechins, the predominant flavonoids in green tea derived from *C. sinensis*, have been associated with various health benefits, including anticancer, anti-obesity, and antiviral activities [81]. The bioavailability and pharmacokinetics of these flavonoids are influenced by their chemical structure and their interaction with other components of the plant matrix [82].

**Figure 2:**
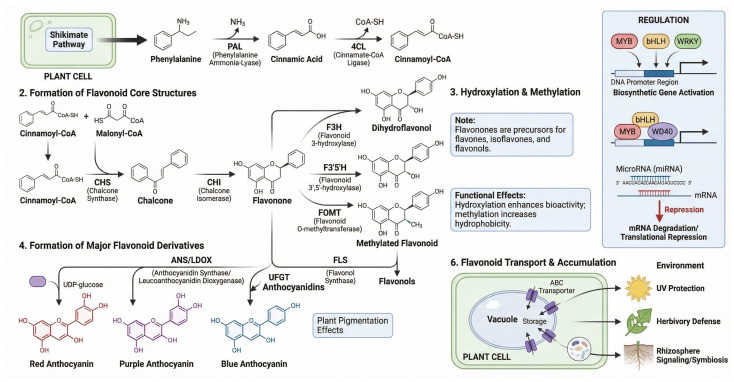
Schematic representation of the flavonoid biosynthesis pathway in medicinal plants. The diagram shows the conversion of phenylpropanoid precursors into major flavonoid classes, including flavones, flavonols, anthocyanins, and related derivatives. Key enzymatic steps, regulatory factors, and transport/accumulation processes are indicated to highlight how flavonoid biosynthesis is controlled and linked to therapeutic bioactivity. Elements in the figure are represented schematically for conceptual illustration only and are not intended to depict complete molecular structures, enzyme kinetics, stoichiometric relationships, regulatory networks, or all intermediate biochemical reactions involved in flavonoid biosynthesis. PAL, phenylalanine ammonia-lyase; 4CL, 4-coumarate:CoA ligase; CHS, chalcone synthase; CHI, chalcone isomerase; F3H, flavonoid 3-hydroxylase; F3′5′H, flavonoid 3′,5′-hydroxylase; FOMT, flavonoid *O*-methyltransferase; FLS, flavonol synthase; ANS/LDOX, anthocyanidin synthase/leucoanthocyanidin dioxygenase; ABC, ATP-binding cassette.

Figure 2 illustrates the flavonoid biosynthesis pathway in medicinal plants, highlighting the key enzymes that convert phenolic precursors into flavonoid compounds and illustrating their associated therapeutic applications.

### 3.2. Alkaloids

Alkaloids are a diverse class of nitrogen-containing compounds with a wide range of biological activities. In plants, these compounds are primarily produced as defense mechanisms against herbivores and pathogens, and many exhibit strong bioactivity [83]. Alkaloid biosynthesis proceeds via several metabolic pathways that originate from amino acid precursors, such as phenylalanine, tyrosine, ornithine, and tryptophan [84]. Depending on their precursor molecules and the enzymes involved, alkaloids are classified into several subgroups, such as indole, isoquinoline, and tropane alkaloids. These compounds have been used medicinally for centuries, with applications ranging from analgesics and sedatives to antimalarial and anticancer agents [85].

One of the most well-known alkaloids is morphine, derived from P. somniferum (opium poppy). Morphine has long been used as a potent analgesic, especially in managing chronic pain and postoperative recovery [86]. The biosynthetic pathway leading to morphine originates from tyrosine-derived intermediates and involves several key enzymes, including tyrosine decarboxylase, which contributes to dopamine formation. Dopamine then condenses with 4-hydroxyphenylacetaldehyde to form (S)-norcoclaurine, which is subsequently converted to (S)-reticuline, the key intermediate in morphine biosynthesis [87]. Functional genomic studies of *P. somniferum* have identified key genes involved in morphine biosynthesis, enabling metabolic engineering approaches to enhance morphine production in non-traditional systems such as microbial platforms and plant cell cultures [88].

Nicotine from *Nicotiana tabacum* functions as a stimulant, while caffeine from *Coffea arabica* and *C. sinensis* exerts stimulatory effects on the central nervous system [89]. Alkaloids such as quinine from *Cinchona officinalis* have long been used to treat malaria, while vincristine and vinblastine from *Catharanthus roseus* are widely used anticancer agents that disrupt mitotic spindle formation in dividing cells [90].

### 3.3. Terpenoids and Essential Oils

Terpenoids, also referred to as isoprenoids, represent the largest class of plant secondary metabolites and are responsible for the distinctive aromas and flavors of many medicinal plants. These compounds are biosynthesized from isoprene units and are classified into several subclasses, including monoterpenes, sesquiterpenes, diterpenes, and triterpenes [91]. Terpenoids play crucial roles in plant defense, communication, and reproduction, often serving as deterrents to herbivores or as attractants for pollinators. Essential oils, which are complex mixtures of terpenoids, exhibit diverse medicinal properties, including antimicrobial, anti-inflammatory, and analgesic activities [92].

**Figure 3:**
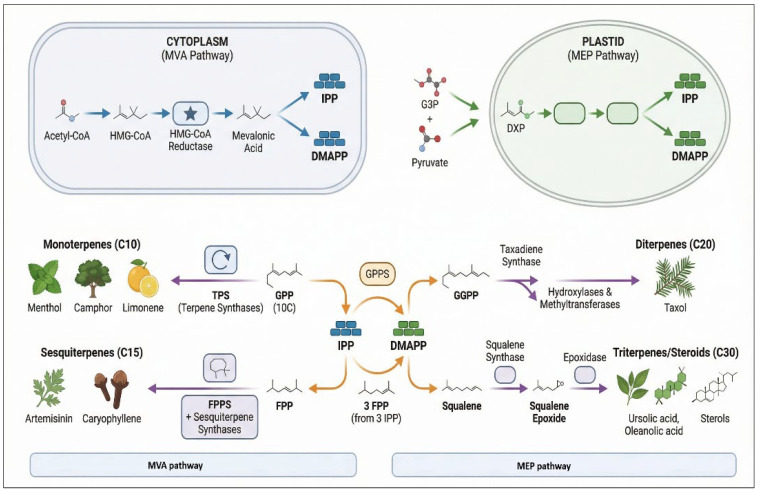
Schematic overview of terpenoid biosynthesis in medicinal plants, showing the cytosolic mevalonate (MVA) pathway and plastidial methylerythritol phosphate (MEP) pathway leading to the formation of isoprenoid precursors (IPP and DMAPP). These intermediates are further converted into various classes of terpenoids, including monoterpenes, sesquiterpenes, diterpenes, and triterpenes/steroids, through key enzymes such as terpene synthases and prenyltransferases. Structures are presented in simplified form for illustrative purposes. MVA, mevalonate pathway; MEP, methylerythritol phosphate pathway; IPP, isopentenyl pyrophosphate; DMAPP, dimethylallyl pyrophosphate; GPP, geranyl pyrophosphate; FPP, farnesyl pyrophosphate; GGPP, geranylgeranyl pyrophosphate; TPS, terpene synthases; GPPS, geranyl pyrophosphate synthase; FPPS, farnesyl pyrophosphate synthase.

A prominent example of terpenoid biosynthesis is the production of limonene, a monoterpene abundant in Citrus fruits. Limonene is widely used for its anti-inflammatory and anticancer properties and is often employed as a natural solvent and flavoring agent [93]. Another significant terpenoid is linalool, present in Lavandula angustifolia (lavender), which exhibits anxiolytic and relaxant effects [94]. Terpenoid biosynthesis in plants is regulated by several enzymes, including terpene synthases, which catalyze the conversion of geranyl pyrophosphate (GPP) into monoterpenes and farnesyl pyrophosphate into sesquiterpenes [95] (Figure 3).

### 3.4. Glycosides

Glycosides represent another important class of secondary metabolites, consisting of a sugar moiety linked to a non-sugar (aglycone) via a glycosidic bond. In plants, glycosides serve as a storage form for bioactive compounds, which can be activated under specific conditions, such as tissue damage [96]. Glycosides are classified based on the type of aglycone present, including cardiac glycosides, flavonoid glycosides, saponins, and anthraquinone glycosides. These compounds exhibit various pharmacological activities, such as anti-inflammatory, antimicrobial, and anticancer effects [97,98].

A representative glycoside group is ginsenosides, the bioactive compounds present in *P. ginseng* [99]. Ginsenosides are primarily responsible for the adaptogenic properties of ginseng, including immune system enhancement, fatigue reduction, and improved cognitive performance [100]. Ginsenosides biosynthesis proceeds through multiple enzymatic steps, beginning with triterpenoid precursors and leading to the formation of various ginsenoside aglycones [101]. Advances in functional genomics have enabled the identification of key enzymes in this pathway, creating opportunities for metabolic engineering to enhance the production of these valuable compounds [102].

Genomic studies of *P. ginseng* have identified several enzymes critical for ginsenoside biosynthesis, including cytochrome P450s and glycosyltransferases [103]. Understanding the genetic basis of ginsenoside production enables the development of transgenic plants or microbial systems with enhanced ginsenoside yields. This knowledge is also essential for improving the medicinal value of ginseng through selective breeding or targeted genetic modification [104].

## 4. Integrating Functional Genomics and Biotechnology for Enhanced Metabolite Production

Integrating functional genomics and biotechnology has created significant opportunities to enhance the production of bioactive compounds in medicinal plants. Combining genomic tools with biotechnological techniques provides a robust framework for optimizing secondary metabolite biosynthesis in medicinal plants through genetic modification or synthetic biology [105]. This approach offers a more efficient and sustainable method for producing valuable metabolites that are otherwise difficult or costly to extract in large quantities from plants [12]. Consequently, combining genetic engineering, metabolic engineering, and synthetic biology is essential for advancing the production of bioactive compounds for pharmaceutical applications [106]. Furthermore, using plant cell cultures and bioreactor systems enables the scalable production of medicinal plant metabolites, creating opportunities for commercial-scale biomanufacturing [107].

### 4.1. Metabolic Engineering for Increased Yields

**Figure 4:**
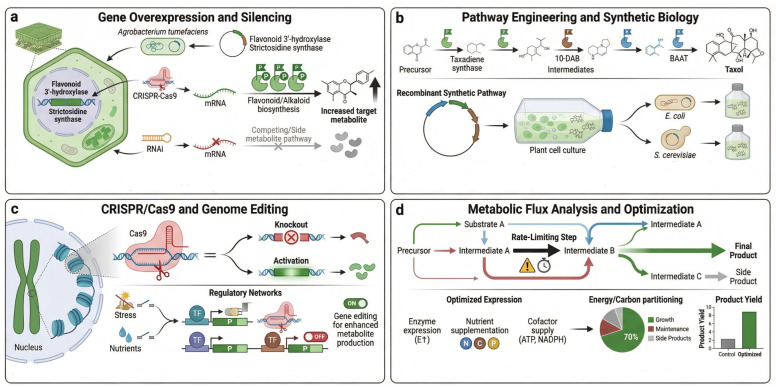
Metabolic engineering strategies in medicinal plants used in current practices. This figure illustrates four common approaches in plant biotechnology and metabolic engineering to optimize the yield of bioactive compounds: (**a**) Gene Overexpression and Silencing: Utilization of Agrobacterium-mediated transformation, CRISPR-Cas9 for activation, and RNAi for silencing to redirect metabolic pathways toward desired products (e.g., flavonoids and alkaloids). (**b**) Pathway Engineering and Synthetic Biology: Reconstruction of complex biosynthetic pathways (like the Taxol pathway) into heterologous hosts such as *E. coli* or *S. cerevisiae* using recombinant DNA technology. (**c**) CRISPR/Cas9 and Genome Editing: Targeted genomic modifications, including gene knockouts and transcriptional activation, regulated by environmental stressors or nutrient availability to enhance metabolite synthesis. (**d**) Metabolic Flux Analysis and Optimization: Identification of rate-limiting steps and optimization of energy/carbon partitioning to maximize product yield while minimizing side products. Elements are represented schematically for conceptual illustration rather than detailed molecular or biochemical mechanisms. BAAT: Baccatin III:benzyl-CoA benzyltransferase; 10-DAB: 10-deacetylbaccatin III (a key intermediate in Taxol biosynthesis); N C P: Nitrogen (N), Carbon (C), and Phosphorus (P) (essential nutrients for metabolic optimization).

Metabolic engineering is a central strategy for optimizing secondary metabolite production in medicinal plants. Modification of plant metabolic pathways increases the yield of specific bioactive compounds, enhances their stability, and promotes the biosynthesis of rare metabolites [108]. A key approach in metabolic engineering is gene overexpression, which boosts the activity of biosynthetic genes to drive higher metabolite production [109]. Another strategy involves pathway optimization, in which key enzymes are manipulated or new biosynthetic genes are introduced to create more efficient pathways for metabolite synthesis [110]. Additionally, using bioreactors enables the scalable production of plant metabolites, allowing high-throughput synthesis under controlled conditions [111]. Microbial host engineering has also been explored for the production of plant-derived metabolites, including artemisinin pathway intermediates [112,113]. Figure 4 illustrates several metabolic engineering strategies for enhancing the yield of medicinal compounds, including gene overexpression, pathway optimization, and the use of bioreactors for large-scale production.

### 4.2. Synthetic Biology for Novel Metabolite Creation

Synthetic biology is an emerging discipline that integrates genomics, biotechnology, and engineering to design and construct novel biological systems or pathways [114]. In medicinal plants, this approach enables the creation of synthetic pathways to produce novel metabolites not naturally present in a plant or to enhance the synthesis of existing bioactive compounds [115]. Reprogramming the metabolic networks of plants or microbes facilitates the establishment of entirely new biosynthetic pathways or the optimization of existing ones to enhance the production of bioactive compounds [106]. Synthetic biology also facilitates the production of metabolites that are otherwise difficult to extract from plants due to low natural abundance or the complexity of their biosynthetic pathways [115].

A notable case study in synthetic biology is the production of Taxol in yeast. Taxol, a potent anticancer drug originally obtained from the bark of the Pacific yew tree (*Taxus brevifolia*), is in short supply [116]. To address this limitation, yeast cells have been engineered to produce Taxol by integrating key biosynthetic genes from *Taxus* spp. into the yeast genome. This synthetic pathway enables large-scale taxol production under controlled and cost-effective conditions [117]. The success of this project highlights the potential of synthetic biology to generate novel metabolites in microorganisms, circumventing the limitations of traditional plant-based extraction methods [118]. Table 2 summarizes key synthetic biology applications in medicinal plant research, showcasing how synthetic pathways have been engineered in plants and microbes to produce novel metabolites.

### 4.3. Plant Cell Cultures and Bioreactors for Large-Scale Production

Plant cell cultures, especially hairy root and suspension cultures, are essential tools for producing bioactive compounds. These cultures enable plant cells to be grown outside the plant, providing a controlled environment for metabolite production [126]. Plant cell cultures are especially useful for producing alkaloids, terpenoids, and glycosides, as they mimic natural biosynthetic pathways in intact plants [127]. A major advantage of plant cell cultures is their maintenance in bioreactors, which provide conditions for high-yield secondary metabolite production [128]. Optimizing bioreactor conditions, including nutrient levels, pH, and temperature, maximizes the production of the desired compounds [129].

The use of plant cell cultures is the production of alkaloids in *Atropa belladonna*. *Atropa belladonna*, or deadly nightshade, is a rich source of tropane alkaloids, including atropine and scopolamine, which are used to treat motion sickness, eye disorders, and Parkinson’s disease [130]. Traditionally, these alkaloids are extracted from the plant, but low cultivation yields make this practice unsustainable. The use of hairy root cultures in bioreactors allows large-scale and more sustainable production of these alkaloids [131]. These plant cell cultures are maintained in controlled environments, allowing optimized alkaloid production without large-scale field cultivation. This example demonstrates how biotechnological tools, including plant cell cultures and bioreactor systems, enable large-scale medicinal plant metabolite production [132]. 

## 5. Case Studies in Medicinal Plants with Advanced Functional Genomics

Functional genomics applied to medicinal plants enhances understanding of the biosynthetic pathways that produce bioactive compounds [133]. The integration of high-throughput sequencing, gene editing, and other molecular techniques facilitates the identification of essential genes and regulatory mechanisms responsible for the biosynthesis of essential metabolites in plants, including artemisinin, cannabinoids, ginsenosides, and taxol [134]. These case studies illustrate how genomics and biotechnology enhance the production and application of valuable medicinal compounds. Integrating these technologies improves yields, enables more sustainable production, and provides new insights into plant metabolism [13].

### 5.1. Artemisia annua (Artemisinin Production)

*A. annua*, commonly known as sweet wormwood, is the primary source of artemisinin, a potent compound used to treat malaria [135]. It is one of the most important drugs, especially in malaria-endemic regions, including Sub-Saharan Africa and Southeast Asia [136]. Artemisinin biosynthesis in *A. annua* involves a complex series of enzymatic steps, beginning with geranylgeranyl pyrophosphate (GGPP) and proceeding through reactions catalyzed by enzymes including artemisinin synthase (CYP71AV1), which converts precursor molecules into artemisinin [137].

Functional genomics studies in *A. annua* have identified essential genes responsible for artemisinin biosynthesis. High-throughput RNA-seq allows mapping of the *A. annua* transcriptome under various growth conditions and facilitates the identification of genes upregulated during artemisinin production. For instance, cytochrome P450 enzymes and GGPS have been identified as central pathway components [138]. Additionally, CRISPR/Cas9 has been applied to manipulate these genes, resulting in increased artemisinin yields in genetically modified plants [139].

**Figure 5:**
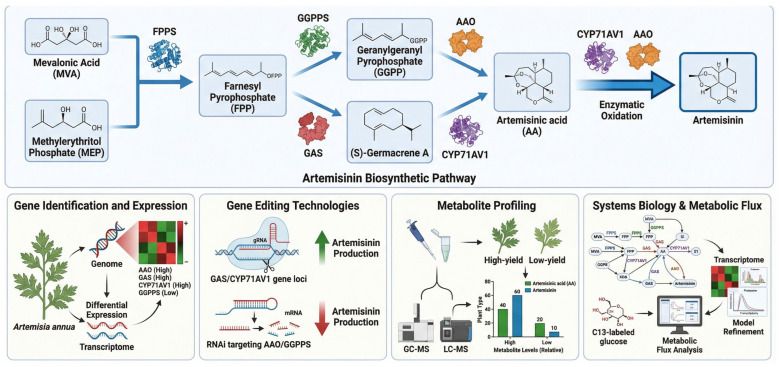
Functional genomics in *Artemisia annua* for artemisinin production. Schematic representation of the Artemisinin Biosynthetic Pathway and the multi-omics strategies used to optimize its production in *A. annua*. The upper panel illustrates the biochemical conversion of primary precursors (MVA and MEP) into artemisinin via key intermediates such as FPP, GGPP, and Artemisinic acid. The lower panels highlight the application of Gene Identification, CRISPR-based Gene Editing, Metabolite Profiling (GC-MS/LC-MS), and Systems Biology (C13-labeled flux analysis) to identify high-yield phenotypes and refine metabolic models for improved yield. Elements are represented schematically for conceptual illustration rather than detailed molecular or biochemical mechanisms. Elements in the figure are represented schematically for conceptual illustration only and are not intended to depict all molecular details, enzyme kinetics, stoichiometric relationships, regulatory complexity, or every intermediate step involved in artemisinin biosynthesis and its associated analytical framework. AA, artemisinic acid; AAO, amorphadiene oxidase; CYP71AV1, cytochrome P450 monooxygenase 71AV1; FPP, farnesyl pyrophosphate; FPPS, farnesyl pyrophosphate synthase; GAS, germacrene A synthase; GGPP, geranylgeranyl pyrophosphate; GGPPS, geranylgeranyl pyrophosphate synthase; MEP, methylerythritol phosphate; MVA, mevalonic acid; RNAi, RNA interference.

A key application of synthetic biology is the engineering of yeast for artemisinin production. Transfer of the biosynthetic pathway from *A. annua* into *S. cerevisiae* (baker’s yeast) has led to the successful development of a microbial platform capable of producing artemisinin in large quantities [140]. This synthetic biology approach enables cost-effective, scalable production, circumventing the limited availability of natural sources [141]. Figure 5 shows the artemisinin biosynthesis pathway in *A. annua*, highlighting key enzymes, artemisinin synthase, cytochrome P450s, and GGPS, and their roles in converting precursor molecules into artemisinin.

### 5.2. Cannabis sativa (Cannabidiol and Tetrahydrocannabinol Production)

The *C. sativa* plant produces cannabinoids, including THC and CBD, which have diverse medicinal and therapeutic properties [142]. THC is mainly responsible for psychoactive effects, whereas CBD provides antianxiety, anti-inflammatory, and analgesic benefits [143]. Cannabinoid biosynthesis in *C. sativa* begins with GPP and olivetolic acid to form the precursor molecule cannabigerol acid (CBGA), which is then converted into THC and CBD [144].

Genomic studies show that the Cannabis genome contains genes encoding major enzymes in cannabinoid biosynthesis, including tetrahydrocannabinolic acid synthase (THCAS) and cannabidiolic acid synthase. These enzymes convert CBGA into THC and CBD, respectively [145]. RNA-seq maps the expression profiles of these genes across various *Cannabis* strains and growth conditions, revealing how genetic variation influences cannabinoid production [146]. Furthermore, genomic diversity in *Cannabis* influences cannabinoid profiles, with some strains producing more CBD and others more THC [147].

A key case study of the genomic approach is the optimization of CBD production in specific *C. sativa* strains [148]. Editing the *THCAS* gene using CRISPR/Cas9 has redirected the biosynthetic pathway toward CBD rather than THC, resulting in the production of high-CBD strains with reduced psychoactive effects [149]. Table 3 compares cannabinoid-producing *Cannabis* strains, highlighting genetic differences underlying THC and CBD variability.

### 5.3. Panax ginseng (Ginsenosides Production)

*P. ginseng*, commonly known as ginseng, is a well-known medicinal plant valued for its adaptogenic properties, which enhance mental performance, reduce stress, and support immune function [157]. The primary bioactive compounds in ginseng are ginsenosides, a class of triterpenoid saponins. Ginsenoside biosynthesis involves cyclizing squalene into a triterpenoid structure, then modifying it with various enzymes to produce the different ginsenosides found in the plant [158]. Ginsenosides are classified into protopanaxadiol and protopanaxatriol, based on their aglycone structures [159].

Functional genomics has clarified the ginsenoside biosynthesis pathway. RNA-seq analyses have identified key genes mediating triterpenoid glycosylation and hydroxylation, both of which are essential for ginsenoside production [160]. For example, cytochrome P450 enzymes and glycosyltransferases modify the aglycone to generate diverse ginsenosides [161]. Additionally, genomic analyses have identified regulatory genes controlling metabolic flux in the pathway. These findings enable metabolic engineering strategies to increase ginsenoside yield in ginseng plants and microbial systems. Overexpression of key ginsenoside pathway genes significantly increases ginsenoside production in plant tissue cultures [162].

### 5.4. Taxus baccata (Taxol Production)

*T. baccata*, commonly known as the yew tree, produces taxol, a key chemotherapy drug for breast, ovarian, and lung cancers [163]. Taxol, a terpenoid, stabilizes microtubules, thereby inhibiting cell division and triggering apoptosis in cancer cells [164]. However, Taxol production in *T. baccata* is limited due to its low concentration in the plant, making bark extraction inefficient and unsustainable [165].

Functional genomics has clarified the taxol biosynthetic pathway in *Taxus* spp., beginning with the precursor GGPP [166]. Available evidence identifies major genes for taxane intermediate synthesis, including taxadiene synthase and taxadiene-5α-hydroxylase, which are essential enzymes in the taxol biosynthesis pathway [167]. Gene expression profiling combined with CRISPR/Cas9-mediated editing has facilitated the engineering of *Taxus* cells and microbial systems for increased taxol production under controlled conditions [168]. Additionally, synthetic biology enables taxol biosynthesis in *E. coli* and *S. cerevisiae*, providing a sustainable, scalable production system. Optimizing taxol production has significant implications for cancer therapy and the commercial production of other terpenoid drugs [169].

### 5.5. Catharanthus roseus (Vinblastine and Vincristine Production)

*Catharanthus roseus* is an important medicinal plant and the major natural source of the anticancer monoterpenoid indole alkaloids vinblastine and vincristine. Functional genomics has substantially advanced understanding of this complex pathway by linking transcriptomic, metabolomic, and regulatory data with alkaloid biosynthesis [170]. Key transcription factors, including BIS1, BIS2, ORCA, and MYC-family regulators, have been shown to control different branches of monoterpenoid indole alkaloid biosynthesis and improve metabolic flux toward valuable alkaloids [171,172]. In addition, pathway elucidation studies have identified important enzymatic steps required for the assembly of vinblastine and vincristine precursors [173]. More recently, single-cell multi-omics has revealed cell-type-specific regulatory networks involved in alkaloid biosynthesis, providing new targets for precision metabolic engineering [174].

### 5.6. Papaver somniferum (Morphine and Codeine Production)

*Papaver somniferum* is the principal commercial source of the benzylisoquinoline alkaloids morphine and codeine. Functional genomics, transcriptomics, metabolomics, and genome-scale analyses have identified key enzymes, gene clusters, and regulatory mechanisms involved in morphinan alkaloid biosynthesis [175]. Comparative transcript and alkaloid profiling identified salutaridine reductase as an important enzyme in morphine biosynthesis, demonstrating the value of gene-to-metabolite approaches in pathway discovery [176]. Genome analysis further revealed that gene clustering and copy number variation strongly influence alkaloid composition and yield in opium poppy [177]. CRISPR/Cas9-mediated editing has also been used to manipulate benzylisoquinoline alkaloid biosynthesis, confirming the potential of genome editing for modifying alkaloid profiles in medicinal plants [178].

## 6. Future Directions in Medicinal Plant Genomics and Biotechnology

Advances in medicinal plant genomics are enabling more efficient and sustainable production of bioactive compounds. Among the most promising innovations are artificial intelligence (AI) and machine learning (ML), which are transforming approaches to gene–metabolite relationships [179]. Rapid advances in genomic sequencing now allow the generation of vast plant genomic datasets. AI and ML can analyze this data to identify genes that regulate metabolite production and their interactions with environmental factors. This influences metabolite optimization, genetic engineering, and predictive modeling in plant biotechnology [180].

### 6.1. Artificial Intelligence and Machine Learning in Plant Genomics

AI and ML are becoming increasingly important tools in plant genomics, particularly for predicting gene–metabolite relationships. These approaches enable the analysis of large and complex datasets generated from RNA-seq, genome-wide association studies, and metabolomic profiling [181]. By applying advanced computational models, AI and ML can identify hidden patterns in gene expression and metabolic regulation associated with the biosynthesis of secondary metabolites in medicinal plants [182]. For example, ML models may help predict how genetic variation influences the production of flavonoids, terpenoids, and alkaloids. This predictive capability can assist in selecting plant lines with improved phytochemical traits and may also support genetic engineering strategies aimed at enhancing the production of target bioactive compounds [183]. However, such predictions should be interpreted cautiously, because model accuracy depends strongly on dataset quality, biological context, and environmental variability. In addition, predicted gene–metabolite relationships still require experimental validation before practical application.

**Figure 6:**
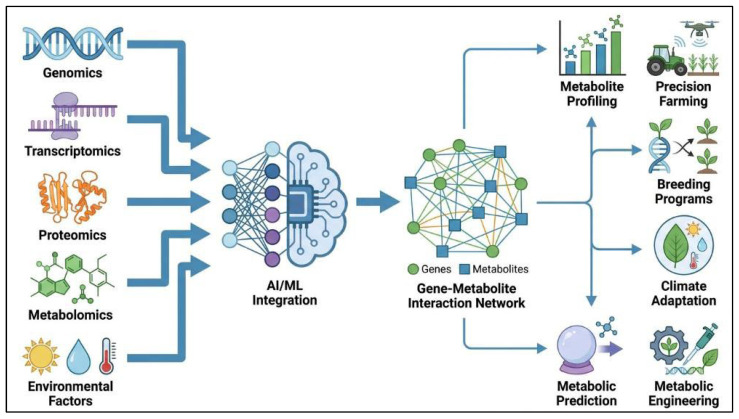
Schematic overview of artificial intelligence (AI) and machine learning (ML) applications in predicting plant metabolite production. Multi-omics data, including genomics, transcriptomics, proteomics, metabolomics, and environmental factors, are integrated through AI/ML approaches to construct gene–metabolite interaction networks. These models enable metabolite profiling and prediction, supporting downstream applications such as precision farming, breeding programs, climate adaptation, and metabolic engineering.

AI can also incorporate environmental variables such as temperature, light intensity, and soil composition into predictive models of metabolite production. By evaluating how these factors influence gene expression and metabolic pathways, AI-based approaches may help optimize cultivation conditions for improved bioactive compound yield [184]. This has potential value in sustainable agriculture, where data-driven approaches can improve crop productivity while reducing environmental impact [185] (Figure 6). Nevertheless, the broader application of AI in medicinal plant biotechnology remains limited by the lack of standardized datasets, incomplete biological annotation, and insufficient validation across diverse plant species and cultivation systems.

Multi-omics integration is especially important because each omics layer provides complementary information. Genomics helps identify candidate biosynthetic genes and regulatory loci, transcriptomics shows when and where these genes are expressed, and metabolomics connects gene activity with the accumulation of specific bioactive compounds [186]. When combined through correlation analysis, pathway mapping, and machine learning models, these datasets can be used to identify candidate gene–metabolite associations, prioritize regulatory genes, and predict metabolic bottlenecks for pathway engineering [179,187]. Therefore, integrated multi-omics provides a systems-level framework for linking molecular discovery with the targeted improvement of medicinal plant metabolites.

### 6.2. Personalized Medicine and Phytochemicals

Personalized medicine is gaining traction in healthcare, with phytochemicals emerging as a potential complementary area of interest. Tailoring the production of specific bioactive compounds based on the genetic makeup and health needs of an individual may eventually contribute to more targeted therapies [188]. Functional genomics and biotechnology play an important role in developing custom phytochemicals targeting specific health conditions, including inflammatory diseases, cardiovascular disorders, or cancer [189].

The integration of genomic data and metabolomic profiling supports the identification of plant-derived bioactive compounds with therapeutic properties potentially aligned with an individual’s genetic profile. For example, flavonoids, including quercetin, may benefit individuals genetically predisposed to cardiovascular disease, while cannabinoids such as CBD may better aid those with anxiety disorders [190]. Genetically modifying plants to produce targeted phytochemicals in higher concentrations or synthesizing them in microorganisms offers a conceptual framework for personalized interventions, but such approaches still require robust pharmacological validation, safety testing, dose standardization, and demonstration of clinical efficacy before healthcare implementation [191].

Moreover, biotechnological innovations, including CRISPR-based gene editing and synthetic biology, are enabling scalable, personalized phytochemical production. For example, genetically modifying *C. sativa* to increase CBD levels could enable personalized treatment for patients with specific mental health conditions [192]. However, translation into clinical practice remains complex because regulatory approval, quality control, long-term safety assessment, and ethical oversight are essential prerequisites for application in healthcare. Accordingly, personalized phytochemical therapies should currently be viewed as a promising but still emerging direction rather than an immediately applicable clinical solution.

### 6.3. Sustainable and Scalable Biotechnologies for Medicinal Plants

Rising demand for medicinal plant compounds calls for sustainable, scalable production systems. Traditional cultivation, though sometimes effective, often cannot meet global demand, especially for rare or high-value metabolites, including artemisinin, taxol, and ginsenosides [193]. The challenge lies in developing cost-effective and environmentally friendly methods for scaling up production without relying on unsustainable harvesting of wild plant populations or large-scale monoculture farming [128].

One solution is using bioreactors and plant cell cultures, which offer a controlled environment for producing medicinal compounds from plant cells or tissues. Hairy root and suspension cell cultures have successfully produced high yields of alkaloids, terpenoids, and glycosides in vitro [194]. Moreover, hydroponics, a soil-free method, enables controlled cultivation of medicinal plants, reducing land use and environmental impact [195]. These biotechnological systems enable consistent production of medicinal compounds, regardless of climate or season.

Green biotechnology, using bioreactors, microbial systems, and genetically engineered plants, is transforming sustainable production of plant-based medicines [196]. The integration of functional genomics with these biotechnological approaches provides a foundation for optimizing plant genetic pathways, thereby improving therapeutic yield and quality while reducing production costs. Furthermore, advances in bioprocessing and scale-up technologies enable the commercialization of large-scale bioactive metabolite production, ensuring a steady supply of plant-based medicines without ecological consequences from over-harvesting [197]. Nevertheless, the field continues to face challenges in fully integrating genomic discovery, multi-omics validation, and scalable production technologies across the broad diversity of medicinal plant species, highlighting important opportunities for further refinement and translation. Despite these advances, the translation of laboratory-scale genomic and synthetic biology breakthroughs into field, clinical, and pharmaceutical settings remains challenging. 

Many engineered traits or enhanced metabolite profiles observed under controlled growth conditions may not be stably maintained under variable field environments because of genotype-by-environment interactions, stress responses, and developmental variation. In addition, metabolite consistency, batch-to-batch reproducibility, and downstream purification remain major obstacles for pharmaceutical applications, where strict quality control, safety, and regulatory standards must be satisfied. Gene-edited or transgenic medicinal plants also require careful evaluation of off-target effects, unintended metabolic alterations, ecological risks, and potential gene flow to wild or cultivated relatives. Regulatory frameworks for genetically modified or gene-edited medicinal plants differ substantially across countries, which can complicate approval, cultivation, commercialization, and clinical translation. In the pharmaceutical context, traceability, product standardization, and long-term safety assessment are also essential. Ethical concerns, including public acceptance, ownership of engineered biological resources, and equitable access to resulting products, further influence the responsible deployment of these technologies. For microbial and cell-culture platforms, additional limitations include pathway instability, low flux efficiency, scale-up costs, and challenges in transferring proof-of-concept systems to industrial bioprocesses. Addressing these translational bottlenecks will require integrated efforts in field validation, regulatory harmonization, process optimization, biosafety assessment, and standardized quality evaluation.

## 7. Conclusions

Integrating functional genomics with biotechnology advances the understanding of plant secondary metabolism, enabling optimized production of bioactive compounds in medicinal plants. This review highlights the latest tools, high-throughput sequencing, CRISPR/Cas9 gene editing, and synthetic biology, and their role in reshaping the production of key metabolites, including artemisinin, cannabinoids, ginsenosides, and taxol. RNA-seq and metagenomics reveal gene-regulatory networks controlling metabolite synthesis, while metabolic engineering redirects plant metabolic pathways to boost valuable compound production. Case studies, *A. annua*, *C. sativa*, *P. ginseng*, and *T. baccata,* show how these technologies and functional genomics enhance plant-based pharmaceutical production.

Innovations in AI and ML are driving an exciting future of medicinal plant biotechnology. These technologies support the prediction of gene–metabolite relationships and the optimization of metabolite production based on genomic and environmental factors. These predictive capabilities support personalized phytochemical production, tailoring medicinal compounds to individual health needs. Furthermore, sustainable biotechnologies, including bioreactors, hydroponics, and plant cell cultures, enable large-scale, efficient, and environmentally friendly production of medicinal compounds. These advancements may reduce reliance on traditional farming methods and provide scalable solutions to meet growing demand for plant-based therapeutics.

In summary, ongoing advances in functional genomics, synthetic biology, and biotechnology promise a bright future for medicinal plant research. These innovations will revolutionize the production and availability of plant-based medicine while enabling sustainable healthcare solutions. Integrating these technologies will help address key challenges in healthcare, agriculture, and environmental sustainability, ensuring that medicinal plants remain a vital source of therapeutics for future generations. Future studies should integrate genomics, transcriptomics, metabolomics, and epigenomics with AI- and ML-based predictive models to improve the identification of gene–metabolite interactions and to uncover regulatory networks governing specialized metabolite biosynthesis. Additional research is also needed to advance personalized phytochemical development by linking plant-derived bioactive compounds with individual therapeutic requirements. At the same time, successful translation of these advances will depend on robust field validation, reproducible metabolite quality, regulatory approval pathways, and economically viable industrial-scale production systems. Moreover, further optimization of sustainable and scalable production platforms, including bioreactors, hydroponics, and plant cell culture systems, will be essential for the efficient and environmentally responsible production of rare and high-value medicinal metabolites.

## Figures and Tables

**Table 1:** List of specialized plant cells and their role in metabolite production.

Specialized Plant Cell/Structure	Typical Location/Anatomy	Major Specialized Metabolite Classes	Role in Biosynthesis/Accumulation of Bioactives	References
Glandular trichomes (capitate/peltate)	Leaves, stems, flowers	Terpenoids, flavonoids, acylsugars	Synthesis, storage, and secretion of bioactives	[51]
Epithelial cells lining secretory cavities (oil glands)	Citrus peel/leaf oil glands	Monoterpenes, coumarins, flavonoids	Essential oil biosynthesis and accumulation	[52]
Secretory cavities (developmental program for “oil glands”)	Internal secretory structures	essential oils, defensive metabolites	Local storage of secondary metabolites	[53]
Resin duct epithelial cells (resin canals/ducts)	Conifer resin canals	Oleoresin terpenoids	Terpenoid synthesis and resin defense	[54]
Laticifers (latex-producing cells/tubes)	Latex-bearing plant tissues	Alkaloids, terpenoids, phenolics	Storage and release of defensive compounds	[55,56]
Phloem-associated laticifers in opium poppy (special case)	Opium poppy phloem region	Benzylisoquinoline alkaloids	Strong example of cell-type	[57,58]
Myrosin idioblasts (“myrosin cells”)	Brassicales tissues near vasculatur	Glucosinolates, myrosinases	Activation of glucosinolate-based defense	[59,60]
Root cortical cells (tobacco; alkaloid-producing root cells)	Tobacco root cortex/tips	Pyridine alkaloids	Root alkaloid biosynthesis and transport	[61,62,63]
Root epidermal/exuding cells (iron-deficiency coumarin system)	Root tissues	Phenylpropanoid-derived coumarins	Roots synthesize and exude coumarins	[64,65]
Epidermal “pigment/phenolic” cells (vacuole-storing epidermis)	Leaf/fruit epidermis;	Flavonols, flavones, anthocyanins	Many flavonoids accumulate predominantly in epidermal	[66,67]
Multicellular compartmentation networks (Catharanthus roseus; pathway split across cell types)	Multiple leaf/stem cell types	Monoterpenoid indole alkaloids	Example of pathway partitioning across multiple cell types + organelles	[68]

**Table 2:** Synthetic biology applications in medicinal plant research.

Application	Synthetic-Biology Approach	Host (Plant/Microbe)	Target Metabolite (s)	References
Production of artemisinin (semisynthetic route)	Artemisinic acid pathway reconstruction and optimization	*Saccharomyces cerevisiae* (yeast)	Artemisinic acid → chemical conversion to artemisinin	[119,120]
Microbial biosynthesis of cannabinoids (de novo and analogs)	Cannabinoid pathway assembly and precursor optimization	*S. cerevisiae* and other microbial hosts	THC, CBD, rare cannabinoids and novel cannabinoid analogs	[121,122]
Engineering flavonoid pathways	Heterologous expression and flux optimization	Microbial hosts (yeast, *E. coli*) and plant cell cultures	Naringenin, baicalin, novel flavonoids	[123,124]
Benzylisoquinoline and other alkaloids in microbes	Reconstruction of plant alkaloid pathways	*E. coli*, *S. cerevisiae*	Morphine precursors, noscapine, berberine, other BIAs	[120,125]
Engineering monoterpene indole alkaloid (MIA) pathways	Gene mining and pathway co-expression	Yeast and plant chassis (including *Nicotiana* transient expression)	MIAs (e.g., vindoline precursors, vinblastine/vincristine precursors)	[119,120]
Production of lignans and other phenylpropanoids in yeast	Cofactor, precursor, and enzyme optimization	*S. cerevisiae*	Podophyllotoxin precursors, lignans, resveratrol	[120]
Plant transient expression and viral vectors for rapid biosynthesis	Viral-vector or transient pathway expression	*N. benthamiana* (plant transient chassis)	VLPs, enzymes for metabolite production, complex proteins	[119]
Expanding isoprenoid chemical space (analog design)	Enzyme engineering and precursor tailoring	Yeast cell factories	Novel monoterpenoids, sesquiterpenoids, cannabinoid analogs	[121]

**Table 3:** Comparison of cannabinoid-producing *Cannabis* strains and their genetic differences.

Chemotype	Typical Cannabinoid Profile	Major Genetic Basis	Biotech & Breeding Strategies	References
High-THC (drug-type)	High Δ9-THC (THCA): low CBD	Functional THCAS; non-functional/low CBDAS	MAS; avoid CBDAS; (CRISPR to modulate expression, research)	[150,151]
High-CBD (hemp)	High CBD (CBDA): very low THC	Functional CBDAS; truncated/nonfunctional THCAS	MAS/genomic selection; CRISPR knockout THCAS (research)	[45,152]
Balanced (THC ≈ CBD)	Similar THCA and CBDA amounts	Heterozygous/combination of THCAS & CBDAS alleles	Marker-guided crosses; cis-regulatory tuning	[153]
CBG-dominant/minor-cannabinoid lines	High CBGA or enriched minor cannabinoids	Loss/reduced activity of downstream synthases or different synthase alleles	Knockout downstream synthases (CRISPR); breed LOF alleles; microbial production	[154,155]
Landrace/diverse germplasm	Broad chemotypic diversity (THC/CBD/minors)	High SNP/CNV diversity at synthase & regulatory loci	Germplasm screening; GWAS; pre-breeding; allele mining	[153,156]

THCAS = THCA synthase; CBDAS = CBDA synthase; CNV = copy number variation; MAS = marker-assisted selection.

## Data Availability

No new data were created or analyzed in this study. Data sharing is not applicable to this article.

## Abbreviations

NGS	Next-generation sequencing
RNA-seq	RNA sequencing
THC	Tetrahydrocannabinol
CBD	Cannabidiol
GPP	Geranyl pyrophosphate
GGPP	Geranylgeranyl pyrophosphate
CBGA	Cannabigerol acid
THCAS	tetrahydrocannabinolic acid synthase
ML	Machine learning

## References

[1] Riaz M., Khalid R., Afzal M., Anjum F., Fatima H., Zia S., Rasool G., Egbuna C., Mtewa A., Uche C. (2023). Phytobioactive compounds as therapeutic agents for human diseases: A review. Food Sci. Nutr..

[2] Atanasov A., Waltenberger B., Pferschy-Wenzig E., Linder T., Wawrosch C., Uhrin P., Temml V., Wang L., Schwaiger S., Heiss E. (2015). Discovery and resupply of pharmacologically active plant-derived natural products: A review. Biotechnol. Adv..

[3] Najmi A., Javed S., Bratty A., Alhazmi H. (2022). Modern Approaches in the Discovery and Development of Plant-Based Natural Products and Their Analogues as Potential Therapeutic Agents. Molecules.

[4] Howes M.J.R., Quave C.L., Collemare J., Tatsis E.C., Twilley D., Lulekal E., Farlow A., Li L., Cazar M.E., Leaman D.J. (2020). Molecules from nature: Reconciling biodiversity conservation and global healthcare imperatives for sustainable use of medicinal plants and fungi. Plants People Planet.

[5] Hussein R.A., El-Anssary A.A. (2019). Plants secondary metabolites: The key drivers of the pharmacological actions of medicinal plants. Herb. Med..

[6] Twaij B.M., Hasan M.N. (2022). Bioactive secondary metabolites from plant sources: Types, synthesis, and their therapeutic uses. Int. J. Plant Biol..

[7] Kaushik B., Sharma J., Kumar P., Shourie A. (2021). Phytochemical properties and pharmacological role of plants: Secondary metabolites. Biosci. Biotechnol. Res. Asia.

[8] Reshi Z.A., Ahmad W., Lukatkin A.S., Javed S.B. (2023). From nature to lab: A review of secondary metabolite biosynthetic pathways, environmental influences, and in vitro approaches. Metabolites.

[9] Singh D., Mathur S., Prasad M., Ranjan R. (2025). Next-generation sequencing in medicinal plants: Recent progress, opportunities, and challenges. J. Plant Growth Regul..

[10] Ho C.H., Piotrowski J., Dixon S.J., Baryshnikova A., Costanzo M., Boone C. (2011). Combining functional genomics and chemical biology to identify targets of bioactive compounds. Curr. Opin. Chem. Biol..

[11] Rynjah D., Sandhanam K., Bhattacharjee B., Deka B., Newar A., Kalita T., Nath J., Ahmed A.B., Sahu R.K., Das T. (2025). CRISPR/Cas9 gene editing systems for enhancing secondary metabolite biosynthesis via reproductive tissue modification. Discov. Plants.

[12] Niazian M. (2019). Application of genetics and biotechnology for improving medicinal plants. Planta.

[13] Devi A.M., Devi K.K., Devi P.P., Devi M.L., Das S. (2023). Metabolic engineering of plant secondary metabolites: Prospects and its technological challenges. Front. Plant Sci..

[14] Bandyopadhyay A., Pal T., Vashisht I. (2026). Pathway Elucidation in Medicinal Plant. Climate Resilience and Molecular Adaptation in Alpine Medicinal Plants: Ecophysiology, Metabolism and Plantomics.

[15] Zhao Y., Liu G., Yang F., Liang Y., Gao Q., Xiang C., Li X., Yang R., Zhang G., Jiang H. (2023). Multilayered regulation of secondary metabolism in medicinal plants. Mol. Hortic..

[16] Benítez-Mateos A.I., Roura Padrosa D., Paradisi F. (2022). Multistep enzyme cascades as a route towards green and sustainable pharmaceutical syntheses. Nat. Chem..

[17] Staniek A., Bouwmeester H., Fraser P.D., Kayser O., Martens S., Tissier A., Van Der Krol S., Wessjohann L., Warzecha H. (2013). Natural products–modifying metabolite pathways in plants. Biotechnol. J..

[18] Yang J., Jia M., Guo J. (2019). Functional genome of medicinal plants. Molecular Pharmacognosy.

[19] Satam H., Joshi K., Mangrolia U., Waghoo S., Zaidi G., Rawool S., Thakare R.P., Banday S., Mishra A.K., Das G. (2023). Next-generation sequencing technology: Current trends and advancements. Biology.

[20] Tyagi P., Singh D., Mathur S., Singh A., Ranjan R. (2022). Upcoming progress of transcriptomics studies on plants: An overview. Front. Plant Sci..

[21] Martin L.B., Fei Z., Giovannoni J.J., Rose J.K. (2013). Catalyzing plant science research with RNA-seq. Front. Plant Sci..

[22] Sharma M.K. (2024). Exploring the biochemical profiles of medicinal plants cultivated under stressful environmental conditions. Curr. Agric. Res. J..

[23] Wang L., Li P., Brutnell T.P. (2010). Exploring plant transcriptomes using ultra high-throughput sequencing. Brief. Funct. Genom..

[24] Guo J., Huang Z., Sun J., Cui X., Liu Y. (2021). Research progress and future development trends in medicinal plant transcriptomics. Front. Plant Sci..

[25] Wu J., Luo Z., Jiang N. (2016). Design of efficient simplified genomic DNA and bisulfite sequencing in large plant populations. Quant. Biol..

[26] Kaur K., Sharma V., Kabila B., Sidhu M.C. (2025). The Science of Omics: A Recent Approach for Screening and Enhancement of Bioactive Compounds in Medicinal Plants. Biotechnological Innovations for Sustainable Biodiversity and Development.

[27] Morris R., Cheng F. (2023). Herbal Medicine and RNA-seq. RNA-Seq in Drug Discovery and Development.

[28] Xu Y., Zhang J., Tang Q., Dai Z., Deng C., Chen Y., Cheng C., Yang Z., Zhang X., Chen J. (2024). Integrated metabolomic and transcriptomic analysis revealed the regulation of yields, cannabinoid, and terpene biosynthesis in *Cannabis sativa* L. under different photoperiods. S. Afr. J. Bot..

[29] Zager J.J., Lange I., Srividya N., Smith A., Lange B.M. (2019). Gene networks underlying cannabinoid and terpenoid accumulation in cannabis. Plant Physiol..

[30] Kim S. (2024). Genetic and Environmental Factors Shaping Cannabis Phenotypes A Study on Temperature Effects and Genetic Regulation of Anthocyanin Accumulation in *Cannabis sativa*. Master’s Thesis.

[31] Sirangelo T.M., Ludlow R.A., Spadafora N.D. (2022). Multi-omics approaches to study molecular mechanisms in *Cannabis sativa*. Plants.

[32] Kumar A., Verma J.P. (2018). Does plant—Microbe interaction confer stress tolerance in plants: A review?. Microbiol. Res..

[33] Agnolucci M., Avio L., Palla M., Sbrana C., Turrini A., Giovannetti M. (2020). Health-promoting properties of plant products: The role of mycorrhizal fungi and associated bacteria. Agronomy.

[34] Sunita K., Mishra I., Mishra J., Prakash J., Arora N.K. (2020). Secondary metabolites from halotolerant plant growth promoting rhizobacteria for ameliorating salinity stress in plants. Front. Microbiol..

[35] Rabiei Z., Hosseini S.J., Pirdashti H., Hazrati S. (2020). Physiological and biochemical traits in coriander affected by plant growth-promoting rhizobacteria under salt stress. Heliyon.

[36] Kapoor R., Anand G., Gupta P., Mandal S. (2017). Insight into the mechanisms of enhanced production of valuable terpenoids by arbuscular mycorrhiza. Phytochem. Rev..

[37] Enebe M.C., Babalola O.O. (2019). The impact of microbes in the orchestration of plants’ resistance to biotic stress: A disease management approach. Appl. Microbiol. Biot..

[38] Nwachukwu B.C., Babalola O.O. (2022). Metagenomics: A tool for exploring key microbiome with the potentials for improving sustainable agriculture. Front. Sustain. Food Syst..

[39] Chen H., Wu H., Yan B., Zhao H., Liu F., Zhang H., Sheng Q., Miao F., Liang Z. (2018). Core microbiome of medicinal plant Salvia miltiorrhiza seed: A rich reservoir of beneficial microbes for secondary metabolism?. Int. J. Mol. Sci..

[40] Rajguru B., Shri M., Bhatt V.D. (2024). Exploring microbial diversity in the rhizosphere: A comprehensive review of metagenomic approaches and their applications. 3 Biotech.

[41] Sharma P. (2025). CRISPR technology: A revolutionary tool in genome editing. Explor. Intersect..

[42] Arora L., Narula A. (2017). Gene editing and crop improvement using CRISPR-Cas9 system. Front. Plant Sci..

[43] Sangeetha V.S., Padmavathi Y.S. (2025). *Artemisia annua*: Illuminating the spectrum of pharmacological wonders. Food Drug Saf..

[44] Li Y., Yang Y., Li L., Tang K., Hao X., Kai G. (2024). Advanced metabolic engineering strategies for increasing artemisinin yield in *Artemisia annua* L.. Hortic. Res..

[45] Deguchi M., Kane S., Potlakayala S., George H., Proano R., Sheri V., Curtis W.R., Rudrabhatla S. (2020). Metabolic engineering strategies of industrial hemp (*Cannabis sativa* L.): A brief review of the advances and challenges. Front. Plant Sci..

[46] Emani C., Ortega A., Duong J., Parungao M., Marti A.F., Anand A. (2025). Genome Engineering and Gene Editing of Cannabis for Crop Improvement. The Cannabis Genome.

[47] Singh S., Praveen A., Dudha N., Sharma V.K., Bhadrecha P. (2024). Single-cell transcriptomics: A new frontier in plant biotechnology research. Plant Cell Rep..

[48] Kim J.-Y., Symeonidi E., Pang T.Y., Denyer T., Weidauer D., Bezrutczyk M., Miras M., Zöllner N., Hartwig T., Wudick M.M. (2021). Distinct identities of leaf phloem cells revealed by single cell transcriptomics. Plant Cell.

[49] Conneely L.J. (2024). *Cannabis sativa* Epigenomics; Regulation of the THCAS and CBDAS Loci. Ph.D. Thesis.

[50] Mansueto L.A. (2024). Resources for *Cannabis sativa* L. Omics Data Analyses. Ph.D. Thesis.

[51] Schuurink R., Tissier A. (2020). Glandular trichomes: Micro-organs with model status?. New Phytol..

[52] Voo S.S., Grimes H.D., Lange B.M. (2012). Assessing the biosynthetic capabilities of secretory glands in Citrus peel. Plant Physiol..

[53] Wang H., Ren J., Zhou S., Duan Y., Zhu C., Chen C., Liu Z., Zheng Q., Xiang S., Xie Z. (2024). Molecular regulation of oil gland development and biosynthesis of essential oils in *Citrus* spp.. Science.

[54] Zulak K.G., Bohlmann J. (2010). Terpenoid biosynthesis and specialized vascular cells of conifer defense. J. Integr. Plant Biol..

[55] Hagel J.M., Yeung E.C., Facchini P.J. (2008). Got milk? The secret life of laticifers. Trends Plant Sci..

[56] Konno K. (2011). Plant latex and other exudates as plant defense systems: Roles of various defense chemicals and proteins contained therein. Phytochemistry.

[57] Ozber N., Carr S.C., Morris J.S., Liang S., Watkins J.L., Caldo K.M., Hagel J.M., Ng K.K.S., Facchini P.J. (2022). Alkaloid binding to opium poppy major latex proteins triggers structural modification and functional aggregation. Nat. Commun..

[58] Lee E.-J., Hagel J., Facchini P. (2013). Role of the phloem in the biochemistry and ecophysiology of benzylisoquinoline alkaloid metabolism. Front. Plant Sci..

[59] Shirakawa M., Tanida M., Ito T. (2022). The Cell Differentiation of Idioblast Myrosin Cells: Similarities with Vascular and Guard Cells. Front. Plant Sci..

[60] Shirakawa M., Hara-Nishimura I. (2018). Specialized Vacuoles of Myrosin Cells: Chemical Defense Strategy in Brassicales Plants. Plant Cell Physiol..

[61] Kato K., Shoji T., Hashimoto T. (2014). Tobacco nicotine uptake permease regulates the expression of a key transcription factor gene in the nicotine biosynthesis pathway. Plant Physiol..

[62] Hildreth S.B., Gehman E.A., Yang H., Lu R.-H., Ritesh K.C., Harich K.C., Yu S., Lin J., Sandoe J.L., Okumoto S. (2011). Tobacco nicotine uptake permease (NUP1) affects alkaloid metabolism. Proc. Natl. Acad. Sci. USA.

[63] Morita M., Shitan N., Sawada K., Van Montagu M.C.E., Inzé D., Rischer H., Goossens A., Oksman-Caldentey K.-M., Moriyama Y., Yazaki K. (2009). Vacuolar transport of nicotine is mediated by a multidrug and toxic compound extrusion (MATE) transporter in *Nicotiana tabacum*. Proc. Natl. Acad. Sci. USA.

[64] Rajniak J., Giehl R.F.H., Chang E., Murgia I., von Wirén N., Sattely E.S. (2018). Biosynthesis of redox-active metabolites in response to iron deficiency in plants. Nat. Chem. Biol..

[65] Ziegler J., Schmidt S., Strehmel N., Scheel D., Abel S. (2017). Arabidopsis Transporter ABCG37/PDR9 contributes primarily highly oxygenated Coumarins to Root Exudation. Sci. Rep..

[66] Ferreyra M.L.F., Serra P., Casati P. (2021). Recent advances on the roles of flavonoids as plant protective molecules after UV and high light exposure. Physiol. Plant.

[67] Passeri V., Koes R., Quattrocchio F.M. (2016). New Challenges for the Design of High Value Plant Products: Stabilization of Anthocyanins in Plant Vacuoles. Front. Plant Sci..

[68] Li F., Shahsavarani M., Handy-Hart C.-J., Côté A., Brasseur-Trottier X., Montgomery V., Beech R.N., Liu L., Bayen S., Qu Y. (2024). Characterization of a vacuolar importer of secologanin in *Catharanthus roseus*. Commun. Biol..

[69] Talei D., Shams A., Khayam Nekouei M. (2025). A Comprehensive Review of Cannabis as a Crucial Pharmaceutical Plant and Its Efficient Propagation Methods. ACS Agric. Sci. Technol..

[70] Depuydt T., De Rybel B., Vandepoele K. (2023). Charting plant gene functions in the multi-omics and single-cell era. Trends Plant Sci..

[71] Patra B., Schluttenhofer C., Wu Y., Pattanaik S., Yuan L. (2013). Transcriptional regulation of secondary metabolite biosynthesis in plants. BBA-Gene Regul. Mech..

[72] Velu G., Palanichamy V., Rajan A.P. (2018). Phytochemical and pharmacological importance of plant secondary metabolites in modern medicine. Bioorganic Phase in Natural Food: An Overview.

[73] Saleem A., Akhtar M.F., Sharif A., Akhtar B., Siddique R., Ashraf G.M., Alghamdi B.S., Alharthy S.A. (2022). Anticancer, cardio-protective and anti-inflammatory potential of natural-sources-derived phenolic acids. Molecules.

[74] Ferrer J.-L., Austin M., Stewart C., Noel J. (2008). Structure and function of enzymes involved in the biosynthesis of phenylpropanoids. Plant Physiol. Biochem..

[75] Vogt T. (2010). Phenylpropanoid biosynthesis. Mol. Plant.

[76] Brodowska K.M. (2017). Natural flavonoids: Classification, potential role, and application of flavonoid analogues. Eur. J. Biol. Res..

[77] Panche A.N., Diwan A.D., Chandra S.R. (2016). Flavonoids: An overview. J. Nutr. Sci..

[78] Bawono L.C., Khairinisa M.A., Jiranusornkul S., Levita J. (2023). The role of catechins of *Camellia sinensis* leaves in modulating antioxidant enzymes: A review and case study. J. Appl. Pharm. Sci..

[79] Musumeci L., Maugeri A., Cirmi S., Lombardo G.E., Russo C., Gangemi S., Calapai G., Navarra M. (2020). Citrus fruits and their flavonoids in inflammatory bowel disease: An overview. Nat. Prod. Res..

[80] Dagher O., Mury P., Thorin-Trescases N., Noly P.E., Thorin E., Carrier M. (2021). Therapeutic potential of quercetin to alleviate endothelial dysfunction in age-related cardiovascular diseases. Front. Cardiovasc. Med..

[81] Musial C., Kuban-Jankowska A., Gorska-Ponikowska M. (2020). Beneficial properties of green tea catechins. Int. J. Mol. Sci..

[82] Zhang J., Wang H., Ai C., Lu R., Chen L., Xiao J., Teng H. (2024). Food matrix-flavonoid interactions and their effect on bioavailability. Crit. Rev. Food Sci. Nutr..

[83] Levin D.A. (1976). The chemical defenses of plants to pathogens and herbivores. Annu. Rev. Ecol. Syst..

[84] Desgagné-Penix I. (2021). Biosynthesis of alkaloids in Amaryllidaceae plants: A review. Phytochem. Rev..

[85] Zandavar H., Babazad M.A. (2023). Secondary Metabolites: Alkaloids. Herbs and Spices: New Advances.

[86] Schiff P.L. (2002). Opium and its alkaloids. Am. J. Pharm. Educ..

[87] Pšenák M. (1999). Biosynthesis of morphinane alkaloids. Poppy.

[88] Halder M., Roy S. (2023). Current status of metabolic engineering of medicinal plants for production of plant-derived secondary metabolites. Medicinal Plants: Biodiversity, Biotechnology and Conservation.

[89] Koltai H., Yaniv Z. (2021). Plants with Phytomolecules Recognized by Receptors in the Central Nervous System. Medicinal Herbs and Fungi: Neurotoxicity vs. Neuroprotection.

[90] Siam M.S.H. (2024). A Review on Synthesis and SARs of Select Natural Alkaloids as Anticancer Agents. Bachelor’s Thesis.

[91] Mabou F.D., Yossa I.B.N. (2021). TERPENES: Structural classification and biological activities. IOSR J. Pharm. Biol. Sci..

[92] Boncan D.A.T., Tsang S.S., Li C., Lee I.H., Lam H.-M., Chan T.-F., Hui J.H. (2020). Terpenes and terpenoids in plants: Interactions with environment and insects. Int. J. Mol. Sci..

[93] Saini R.K., Ranjit A., Sharma K., Prasad P., Shang X., Gowda K.G.M., Keum Y.-S. (2022). Bioactive compounds of citrus fruits: A review of composition and health benefits of carotenoids, flavonoids, limonoids, and terpenes. Antioxidants.

[94] López V., Nielsen B., Solas M., Ramírez M.J., Jäger A.K. (2017). Exploring pharmacological mechanisms of lavender (*Lavandula angustifolia*) essential oil on central nervous system targets. Front. Pharmacol..

[95] Böttger A., Vothknecht U., Bolle C., Wolf A. (2018). Terpenes and terpenoids. Lessons on Caffeine, Cannabis & Co: Plant-Derived Drugs and Their Interaction with Human Receptors.

[96] Alamgir A. (2018). Secondary metabolites: Secondary metabolic products consisting of C and H; C, H, and O; N, S, and P elements; and O/N heterocycles. Therapeutic Use of Medicinal Plants and Their Extracts: Volume 2: Phytochemistry and Bioactive Compounds.

[97] Kowsalya K., Vidya N., Halka J., Preetha J.S.Y., Saradhadevi M., Sahayarayan J.J., Gurusaravanan P., Arun M. (2025). Plant glycosides and glycosidases: Classification, sources, and therapeutic insights in current medicine. Glycoconj. J..

[98] More S., Sawarkar K., Mendhi S., Landge A., Jadhav Y., Sawandhkar P. (2023). A Review on Pharmacognostic and Pharmacological Effects and Action of Anthracene, Cardiac, and Saponin Glycosides. Int. J. Pharm. Sci. Rev. Res..

[99] Ru W., Wang D., Xu Y., He X., Sun Y.-E., Qian L., Zhou X., Qin Y. (2015). Chemical constituents and bioactivities of *Panax ginseng* (CA Mey.). Drug Discov. Ther..

[100] Ratan Z.A., Youn S.H., Kwak Y.-S., Han C.-K., Haidere M.F., Kim J.K., Min H., Jung Y.-J., Hosseinzadeh H., Hyun S.H. (2021). Adaptogenic effects of *Panax ginseng* on modulation of immune functions. J. Ginseng Res..

[101] Yang J.-L., Hu Z.-F., Zhang T.-T., Gu A.-D., Gong T., Zhu P. (2018). Progress on the studies of the key enzymes of ginsenoside biosynthesis. Molecules.

[102] Li M., Ma M., Wu Z., Liang X., Zheng Q., Li D., An T., Wang G. (2023). Advances in the biosynthesis and metabolic engineering of rare ginsenosides. Appl. Microbiol. Biot..

[103] Xue Y., Zhang R., Li T., Deng Q., Luo W., Chang R., Zeng D., Tan J., Sun T., Liu Y.-G. (2025). Sustainable production of ginsenosides: Advances in biosynthesis and metabolic engineering. Plants.

[104] Son S.-H., Kang J., Shin Y., Lee C., Sung B.H., Lee J.Y., Lee W. (2024). Sustainable production of natural products using synthetic biology: Ginsenosides. J. Ginseng Res..

[105] Yamazaki M., Rai A., Yoshimoto N., Saito K. (2018). Perspective: Functional genomics towards new biotechnology in medicinal plants. Plant Biotechnol. Rep..

[106] Sharma M., Koul A., Sharma D., Kaul S., Swamy M.K., Dhar M.K. (2019). Metabolic engineering strategies for enhancing the production of bio-active compounds from medicinal plants. Natural Bio-Active Compounds: Volume 3: Biotechnology, Bioengineering, and Molecular Approaches.

[107] Das S., Manna D., Mondal T., Mondal P. (2025). Advanced Systems and Bioreactors for Large-Scale Secondary Metabolite Production in Medicinal Plants. Biotechnology, Multiple Omics, and Precision Breeding in Medicinal Plants.

[108] Sethi A., Bhandawat A., Pati P.K. (2022). Engineering medicinal plant-derived CYPs: A promising strategy for production of high-valued secondary metabolites. Planta.

[109] Chae T.U., Choi S.Y., Kim J.W., Ko Y.-S., Lee S.Y. (2017). Recent advances in systems metabolic engineering tools and strategies. Curr. Opin. Biotech..

[110] Courchesne N.M.D., Parisien A., Wang B., Lan C.Q. (2009). Enhancement of lipid production using biochemical, genetic and transcription factor engineering approaches. J. Biotechnol..

[111] Abdi G., Tendulkar R., Thatte C., Mishra S., Desai B., Surve S., Chaudhari A., Patil N., Jain M., Tarighat M.A. (2024). Scaling up nature’s chemistry: A guide to industrial production of valuable metabolites. Advances in Metabolomics.

[112] Zeng Q., Qiu F., Yuan L. (2008). Production of artemisinin by genetically-modified microbes. Biotechnol. Lett..

[113] Ferreira J.F., Laughlin J., Delabays N., de Magalhães P.M. (2005). Cultivation and genetics of *Artemisia annua* L. for increased production of the antimalarial artemisinin. Plant Genet. Resour..

[114] Heinemann M., Panke S. (2006). Synthetic biology—Putting engineering into biology. Bioinformatics.

[115] Cravens A., Payne J., Smolke C.D. (2019). Synthetic biology strategies for microbial biosynthesis of plant natural products. Nat. Commun..

[116] Suffness M., Wall M.E. (2021). Discovery and development of taxol. Taxol.

[117] Engels B., Dahm P., Jennewein S. (2008). Metabolic engineering of taxadiene biosynthesis in yeast as a first step towards Taxol (Paclitaxel) production. Metab. Eng..

[118] Qin K., Liu F., Zhang C., Deng R., Fernie A.R., Zhang Y. (2025). Systems and synthetic biology for plant natural product pathway elucidation. Cell Rep..

[119] Osbourn A.E., O’Maille P.E., Rosser S.J., Lindsey K. (2012). Synthetic biology. New Phytol..

[120] Perrin J., Besseau S., Papon N., Courdavault V. (2022). Boosting lignan-precursor synthesis in yeast cell factories through co-factor supply optimization. Front. Bioeng. Biotechnol..

[121] Wang L., Rosenfeldt M., Koutsaviti A., Harizani M., Zhao Y., Leelahakorn N., Frachon A., Raadam M.H., Miettinen K., Pateraki I. (2025). Systematic biotechnological production of isoprenoid analogs with bespoke carbon skeletons. Nat. Commun..

[122] Yan C., Okorafor I.C., Johnson C.W., Houk K.N., Garg N.K., Tang Y. (2025). Microbial biosynthesis of rare cannabinoids. J. Ind. Microbiol. Biotechnol..

[123] Trantas E.A., Koffas M.A.G., Xu P., Ververidis F. (2015). When plants produce not enough or at all: Metabolic engineering of flavonoids in microbial hosts. Front. Plant Sci..

[124] Huang W., Wang Y., Tian W., Cui X., Tu P., Li J., Shi S., Liu X. (2022). Biosynthesis Investigations of Terpenoid, Alkaloid, and Flavonoid Antimicrobial Agents Derived from Medicinal Plants. Antibiotics.

[125] Minami H. (2013). Fermentative Production of Plant Benzylisoquinoline Alkaloids in Microbes. Biosci. Biotechnol. Biochem..

[126] Bapat V.A., Kavi Kishor P., Jalaja N., Jain S.M., Penna S. (2023). Plant cell cultures: Biofactories for the production of bioactive compounds. Agronomy.

[127] Roberts S.C. (2007). Production and engineering of terpenoids in plant cell culture. Nat. Chem. Biol..

[128] Ozyigit I., Dogan I., Hocaoglu-Ozyigit A., Yalçın B., Erdoğan A., Yalcin I., Cabi E., Kaya Y. (2023). Production of secondary metabolites using tissue culture-based biotechnological applications. Front. Plant Sci..

[129] Varma V. (2010). Advancements in the production of secondary metabolites. J. Nat. Prod..

[130] Kwakye G.F., Jiménez J., Jiménez J.A., Aschner M. (2018). Atropa belladonna neurotoxicity: Implications to neurological disorders. Food Chem. Toxicol..

[131] Mishra B.N., Ranjan R. (2008). Growth of hairy-root cultures in various bioreactors for the production of secondary metabolites. Biotechnol. Appl. Bioc.

[132] Mawale K.S., Mahapatra A.S., Pakala H., Giridhar P., Sharma A., Rao N.N. (2025). Tropane Alkaloids In Vitro Production, Current Status, and Perspectives. Tropane Alkaloids: Sources, Chemistry, Pharmacology and Biotechnology.

[133] Younessi-Hamzekhanlu M., Ozturk M., Jafarpour P., Mahna N. (2022). Exploitation of next generation sequencing technologies for unraveling metabolic pathways in medicinal plants: A concise review. Ind. Crops Prod..

[134] Zhao C., Zhang Z., Sun L., Bai R., Wang L., Chen S. (2023). Genome sequencing provides potential strategies for drug discovery and synthesis. Acupunct. Herb. Med..

[135] Xu F., Shan X., Li J., Li J., Yuan J., Zou D., Wang M. (2025). The plant matrix of *Artemisia annua* L. for the treatment of malaria: Pharmacodynamic and pharmacokinetic studies. PLoS ONE.

[136] Rosenthal P., Asua V., Conrad M. (2024). Emergence, transmission dynamics and mechanisms of artemisinin partial resistance in malaria parasites in Africa. Nat. Rev. Microbiol..

[137] Judd R., Bagley M., Li M., Zhu Y., Lei C., Yuzuak S., Ekelöf M., Pu G., Zhao X., Muddiman D. (2019). Artemisinin Biosynthesis in Non-glandular Trichome Cells of *Artemisia annua*. Mol. Plant.

[138] Khan S., Ali A., Saifi M., Saxena P., Ahlawat S., Abdin M. (2020). Identification and the potential involvement of miRNAs in the regulation of artemisinin biosynthesis in A. annua. Sci. Rep..

[139] Koerniati S., Simanjuntak G. (2020). CRISPR/Cas9 system for disruption of biochemical pathway for sterol synthesis in *Artemisia annua* L.. IOP Conf. Ser. Earth Environ. Sci..

[140] Paddon C., Westfall P., Pitera D., Benjamin K., Fisher K., McPhee D., Leavell M., Tai A., Main A., Eng D. (2013). High-level semi-synthetic production of the potent antimalarial artemisinin. Nature.

[141] Ro D., Paradise E., Ouellet M., Fisher K., Newman K., Ndungu J., Ho K., Eachus R., Ham T., Kirby J. (2006). Production of the antimalarial drug precursor artemisinic acid in engineered yeast. Nature.

[142] Kumar P., Mahato D., Kamle M., Borah R., Sharma B., Pandhi S., Tripathi V., Yadav H., Devi S., Patil U. (2021). Pharmacological properties, therapeutic potential, and legal status of *Cannabis sativa* L.: An overview. Phytother. Res..

[143] Sharpe L., Sinclair J., Kramer A., De Manincor M., Sarris J. (2020). Cannabis, a cause for anxiety? A critical appraisal of the anxiogenic and anxiolytic properties. J. Transl. Med..

[144] Luo X., Reiter M., d’Espaux L., Wong J., Denby C., Lechner A., Zhang Y., Grzybowski A., Harth S., Lin W. (2019). Complete biosynthesis of cannabinoids and their unnatural analogues in yeast. Nature.

[145] Grassa C., Weiblen G., Wenger J., Dabney C., Poplawski S., Motley S., Michael T., Schwartz C. (2021). A new Cannabis genome assembly associates elevated cannabidiol (CBD) with hemp introgressed into marijuana. New Phytol..

[146] Sng B.J.R., Jeong Y.J., Leong S.H., Jeong J.C., Lee J., Rajani S., Kim C.Y., Jang I.-C. (2024). Genome-wide identification of cannabinoid biosynthesis genes in non-drug type Cannabis (*Cannabis sativa* L.) cultivar. J. Cannabis Res..

[147] Laverty K., Stout J., Sullivan M., Shah H., Gill N., Holbrook L., Deikus G., Sebra R., Hughes T., Page J. (2018). A physical and genetic map of *Cannabis sativa* identifies extensive rearrangements at the THC/CBD acid synthase loci. Genome Res..

[148] Bitežnik L., Štukelj R., Flajšman M. (2024). The Efficiency of CBD Production Using Grafted *Cannabis sativa* L. Plants Is Highly Dependent on the Type of Rootstock: A Study. Plants.

[149] Deguchi M., Dhir S., Potlakayala S., Dhir S., Curtis W., Rudrabhatla S. (2022). In planta Female Flower Agroinfiltration Alters the Cannabinoid Composition in Industrial Hemp (*Cannabis sativa* L.). Front. Plant Sci..

[150] Xie Z., Mi Y., Kong L., Gao M., Chen S., Chen W., Meng X., Sun W., Chen S., Xu Z. (2023). *Cannabis sativa*: Origin and history, glandular trichome development, and cannabinoid biosynthesis. Hortic. Res..

[151] Oultram J.M.J., Pegler J.L., Bowser T.A., Ney L.J., Eamens A.L., Grof C.P.L. (2021). *Cannabis sativa*: Interdisciplinary Strategies and Avenues for Medical and Commercial Progression Outside of CBD and THC. Biomedicines.

[152] Li L., Yu S., Chen J., Cheng C., Sun J., Xu Y., Deng C., Dai Z., Yang Z., Chen X. (2022). Releasing the Full Potential of Cannabis through Biotechnology. Agronomy.

[153] Enríquez D.J., Alonso J.C., Hille L., Brand S., Holzgrabe U., Vergara D., Montoya G., Ramírez Y.A. (2025). Unveiling Colombia’s medicinal *Cannabis sativa* treasure trove: Phenotypic and Chemotypic diversity in legal cultivation. Phytochem. Anal..

[154] Caprioglio D., Amin H.I.M., Taglialatela-Scafati O., Muñoz E., Appendino G. (2022). Minor Phytocannabinoids: A Misleading Name but a Promising Opportunity for Biomedical Research. Biomolecules.

[155] Wang F., Zang Z., Zhao Q., Xiaoyang C., Lei X., Wang Y., Ma Y., Cao R., Song X., Tang L. (2024). Advancement of Research Progress on Synthesis Mechanism of Cannabidiol (CBD). ACS Synth. Biol..

[156] Laaboudi F.-Z., Rejdali M., Amhamdi H., Salhi A., Elyoussfi A., Ahari M.h. (2024). In the weeds: A comprehensive review of cannabis; its chemical complexity, biosynthesis, and healing abilities. Toxicol. Rep..

[157] Sutopo N.C., Qomaladewi N.P., Lee H., Lee M.S., Kim J.H., Cho J.Y. (2025). Comprehensive understanding and underlying molecular mechanisms of the adaptogenic effects of *Panax ginseng*. J. Ginseng Res..

[158] Han J., In J., Kwon Y., Choi Y.E. (2009). Regulation of ginsenoside and phytosterol biosynthesis by RNA interferences of squalene epoxidase gene in *Panax ginseng*. Phytochemistry.

[159] Hou M., Wang R., Zhao S., Wang Z. (2021). Ginsenosides in Panax genus and their biosynthesis. Acta Pharm. Sin. B.

[160] Hou M., Nie F., Zhao J., Ju Z., Yang L., Wang Q., Zhao S., Wang Z. (2022). New Glycosyltransferases in Panax notoginseng Perfect Main Ginsenosides Biosynthetic Pathways. J. Agric. Food Chem..

[161] Han J., Kim H.-J., Kwon Y., Choi Y.E. (2011). The Cyt P450 enzyme CYP716A47 catalyzes the formation of protopanaxadiol from dammarenediol-II during ginsenoside biosynthesis in *Panax ginseng*. Plant Cell Physiol..

[162] Yao L., Zhang H., Liu Y., Ji Q., Xie J., Zhang R., Huang L., Mei K., Wang J., Gao W. (2022). Engineering of triterpene metabolism and overexpression of the lignin biosynthesis gene PAL promotes ginsenoside Rg3 accumulation in ginseng plant chassis. J. Integr. Plant Biol..

[163] Zhoulideh Y., Mohammadi Y., Mashayekhi M. (2023). The Stimulation of the Anticancer Chemical Taxol in *Taxus baccata*. For. Prod. J..

[164] Lim P., Goh B., Lee W.-L. (2021). Taxol: Mechanisms of action against cancer, an update with current research. Paclitaxel.

[165] Malik S., Cusido R., Mirjalili M., Moyano E., Palazón J., Bonfill M. (2010). Production of the anticancer drug taxol in *Taxus baccata* suspension cultures: A review. Process Biochem..

[166] Croteau R., Ketchum R., Long R., Kaspera R., Wildung M. (2006). Taxol Biosynthesis and Molecular Genetics. Phytochem. Rev..

[167] Sarmadi M., Karimi N., Mirjalili M., Ghassempour A., Cusido R., Palazon J. (2025). Osmotic stress modulates physio-biochemical traits and the expression of key genes involved in taxane biosynthesis in *Taxus baccata* L. cell cultures. Plant Stress..

[168] Sun X., Zhang H., Jia Y., Li J., Jia M. (2024). CRISPR-Cas9-based genome-editing technologies in engineering bacteria for the production of plant-derived terpenoids. Eng. Microbiol..

[169] Ajikumar P., Xiao W., Tyo K., Wang Y., Simeon F., Leonard E., Mucha O., Phon T.H., Pfeifer B., Stephanopoulos G. (2010). Isoprenoid Pathway Optimization for Taxol Precursor Overproduction in *Escherichia coli*. Science.

[170] Rischer H., Orešič M., Seppänen-Laakso T., Katajamaa M., Lammertyn F., Ardiles-Diaz W., Van Montagu M., Inzé D., Oksman-Caldentey K., Goossens A. (2006). Gene-to-metabolite networks for terpenoid indole alkaloid biosynthesis in *Catharanthus roseus* cells. Proc. Natl. Acad. Sci. USA.

[171] Van Moerkercke A., Steensma P., Schweizer F., Pollier J., Gariboldi I., Payne R., Vanden Bossche R., Miettinen K., Espoz J., Purnama P. (2015). The bHLH transcription factor BIS1 controls the iridoid branch of the monoterpenoid indole alkaloid pathway in *Catharanthus roseus*. Proc. Natl. Acad. Sci. USA.

[172] Schweizer F., Colinas M., Pollier J., Van Moerkercke A., Vanden Bossche R., De Clercq R., Goossens A. (2018). An engineered combinatorial module of transcription factors boosts production of monoterpenoid indole alkaloids in *Catharanthus roseus*. Metab. Eng..

[173] Qu Y., Safonova O., De Luca V. (2018). Completion of the canonical pathway for assembly of anticancer drugs vincristine/vinblastine in *Catharanthus roseus*. Plant J..

[174] Li C., Colinas M., Wood J.C., Vaillancourt B., Hamilton J.P., Jones S.L., Caputi L., O’Connor S.E., Buell C.R. (2025). Cell-type-aware regulatory landscapes governing monoterpene indole alkaloid biosynthesis in the medicinal plant *Catharanthus roseus*. New Phytol..

[175] Beaudoin G., Facchini P. (2014). Benzylisoquinoline alkaloid biosynthesis in opium poppy. Planta.

[176] Ziegler J., Voigtländer S., Schmidt J., Kramell R., Miersch O., Ammer C., Gesell A., Kutchan T. (2006). Comparative transcript and alkaloid profiling in *Papaver* species identifies a short chain dehydrogenase/reductase involved in morphine biosynthesis. Plant J. Cell Mol. Biol..

[177] Li Q., Ramasamy S., Singh P., Hagel J., Dunemann S., Chen X., Chen R., Yu L., Tucker J., Facchini P. (2020). Gene clustering and copy number variation in alkaloid metabolic pathways of opium poppy. Nat. Commun..

[178] Alagoz Y., Gurkok T., Zhang B., Unver T. (2016). Manipulating the Biosynthesis of Bioactive Compound Alkaloids for Next-Generation Metabolic Engineering in Opium Poppy Using CRISPR-Cas 9 Genome Editing Technology. Sci. Rep..

[179] Bai W., Li C., Li W., Wang H., Han X., Wang P., Wang L. (2024). Machine learning assists prediction of genes responsible for plant specialized metabolite biosynthesis by integrating multi-omics data. BMC Genom..

[180] Moore B., Wang P., Fan P., Leong B., Schenck C., Lloyd J., Lehti-Shiu M., Last R., Pichersky E., Shiu S.-H. (2018). Robust predictions of specialized metabolism genes through machine learning. Proc. Natl. Acad. Sci. USA.

[181] Asim M., Ibrahim M.A., Asif T., Dengel A. (2025). RNA sequence analysis landscape: A comprehensive review of task types, databases, datasets, word embedding methods, and language models. Heliyon.

[182] Reel P., Reel S., Pearson E., Trucco E., Jefferson E. (2021). Using machine learning approaches for multi-omics data analysis: A review. Biotechnol. Adv..

[183] García-Pérez P., Zhang L., Miras-Moreno B., Lozano-Milo E., Landín M., Lucini L., Gallego P. (2021). The Combination of Untargeted Metabolomics and Machine Learning Predicts the Biosynthesis of Phenolic Compounds in Bryophyllum Medicinal Plants (*Genus kalanchoe*). Plants.

[184] Helmy M., Smith D., Selvarajoo K. (2020). Systems biology approaches integrated with artificial intelligence for optimized metabolic engineering. Metab. Eng. Commun..

[185] Suthers P., Foster C., Sarkar D., Wang L., Maranas C. (2020). Recent advances in constraint and machine learning-based metabolic modeling by leveraging stoichiometric balances, thermodynamic feasibility and kinetic law formalisms. Metab. Eng..

[186] Yang L., Yang Y., Huang L., Cui X., Liu Y. (2022). From single- to multi-omics: Future research trends in medicinal plants. Brief. Bioinform..

[187] Zhang W., Zeng Y., Jiao M., Ye C., Li Y., Liu C., Wang J. (2023). Integration of high-throughput omics technologies in medicinal plant research: The new era of natural drug discovery. Front. Plant Sci..

[188] Sadee W., Wang D., Hartmann K., Toland A. (2023). Pharmacogenomics: Driving Personalized Medicine. Pharmacol. Rev..

[189] Saito K. (2025). Development of phytochemical genomics: From decoding metabolome to functional genomics and biotechnology of plant metabolism. Proc. Jpn. Acad. B-Phys..

[190] Micek A., Godos J., Rio D., Galvano F., Grosso G. (2021). Dietary Flavonoids and Cardiovascular Disease: A Comprehensive Dose-Response Meta-Analysis. Mol. Nutr. Food Res..

[191] Nielsen E., Temporiti M.E.E., Cella R. (2019). Improvement of phytochemical production by plant cells and organ culture and by genetic engineering. Plant Cell Rep..

[192] Parsons J., Martin S., James T., Golenia G., Boudko E., Hepworth S. (2019). Polyploidization for the Genetic Improvement of *Cannabis sativa*. Front. Plant Sci..

[193] Selwal N., Rahayu F., Herwati A., Latifah E., Supriyono, Suhara C., Suastika I.B.K., Mahayu W., Wani A. (2023). Enhancing secondary metabolite production in plants: Exploring traditional and modern strategies. J. Agric. Food Res..

[194] Motolinía-Alcántara E., Castillo-Araiza C., Rodríguez-Monroy M., Román-Guerrero A., Cruz-Sosa F. (2021). Engineering Considerations to Produce Bioactive Compounds from Plant Cell Suspension Culture in Bioreactors. Plants.

[195] Atherton H., Li P. (2023). Hydroponic Cultivation of Medicinal Plants—Plant Organs and Hydroponic Systems: Techniques and Trends. Horticulturae.

[196] Marchev A., Yordanova Z., Georgiev M. (2020). Green (cell) factories for advanced production of plant secondary metabolites. Crit. Rev. Biotechnol..

[197] Bautista-Vanegas F.E., Diaz-Guerrero J.L., Cabezas-Soliz I.N., Apaza-Huanca B., Fernández E.E.V., Auza-Santiváñez J.C., Gamboa T.K.O., Rosales R.R.G., Gómez L.M.T., Carías P. (2025). Bioprocess Engineering: Advances in Cell Culture Systems, Reactor Design, Scale-Up Strategies, and Intensification Processes for the Production of Biological and Bioactive Compounds. eVitroKhem.

